# On the Treatment of Acne*Read before the Eastern Medical Society, New York, February 12, 1897.

**Published:** 1897-04

**Authors:** William S. Gottheil

**Affiliations:** New York, Professor of Dermatology, New York School of Clinical Medicine


### ON THE TREATMENT OF ACNE.*

By WILLIAM S. GOTTHEIL, M.D., New York,
Professor of Dermatology, New York School of Clinical Medicine.

It is our good medical custom to decry routine treatment and to claim an ideal eclecticism that shall individualize each case and select its appropriate treatment from the entire range of therapeutics. But here, as in so many other cases, practice and theory are necessarily at variance. The field of medicine is too large for any one to get more than a bird's-eye view of the whole of it; and a detailed knowledge of minute points is an impossibility for the practitioner. Classification and schematization are, therefore, an absolute necessity, if our knowledge is not to be a jumble of unconnected details. General plans of treatment, routine plans, if you will, are a necessary part of these processes; and they are the only means by which the busy practitioner can arm himself to face the ever varying emergencies of his work.

In a few, a very few conditions, there are generally accepted and simple plans of treatment. In most of them there are as many methods as there are teachers, and only too often do we find, to our sorrow, that the method that is most highly vaunted gives good results only in the hands of its proposer. The practitioner has not the time to experiment, nor can he let his patient's and his own material interests suffer whilst he tries this, that, or the other plan. It is therefore, let me hope, a labor of some utility, when one whose special line of work has given him the opportunity to make the trials, puts in the necessary routine form the results that he has obtained. More especially is this the case when the malady is a common one, that all of us must treat, and an obstinate one, that all of us sometimes fail to cure.

Acne is preëminently such a malady. So common that it is, perhaps, after eczema, the most frequent of dermal affections, and so obstinate that it is occasionally the despair of our therapeutists, it merits our careful consideration. Innumerable are the drugs

*Read before the Eastern Medical Society, New York, February 12, 1897.

that have been recommended, the applications that have been employed. Yet there is no generally applicable and successful treatment for it, or sarsaparilla and blood-purifiers and quack skin remedies would not enjoy the vogue they do.

Acne is partly a disease and partly a physiological process. It is a physiological process inasmuch as it is to some extent a normal accompaniment of the changes that occur in the body at puberty. There is an intimate connection between the skin and the genital system. When the latter begin to undergo the changes of maturity important alterations appear in the integument. The growth of hair on the genitals, the face, and in the axillæ in man, the sprouting of the antlers in the stag, the new and varied plumage of the birds; all these are the external accompaniments of the development of the mammæ, ovaries, and testicles. The sebaceous glands participate in the process; increased sebaceous secretion always occurs; and a slight increase over this normal amount gives comedo, seborrhea, and acne. So common is such an excess that it is not too much to say that twenty-five per cent. of all adolescents suffer more or less from these affections. With the completion of development the excessive action ceases and the maladies in question are rare after the twenty-fifth, and almost unknown after the thirtieth year. The texture of the skin is apparently a not unimportant factor in the development of these cases, and we find the worst ones in persons of dark complexions, with greasy skins, large sebaceous glands with patulous mouths, and coarse hair.

Slight cases of acne are trivial affections, and even severe ones are not serious from a medical point of view. But the disease has psychic, and, if I may be permitted to say so, economic results entirely out of proportion with its effects on the general health of the individual. Occurring, as it does, at a period of life when personal appearance is of the greatest importance, it may be in young girls quite a serious affection. I have under my care at this moment a young lady, accomplished and not ill-favored apart from her affliction, whose face is deformed with comedones, papules, pustules, dermic abscesses and scars, and whose skin exhales an odor of decomposed fat sufficiently pronounced to render her an object of repulsion to all who approach her. She is a recluse from society on account of her affection; her chances of marriage are fatally vitiated; and her life and that of her family are to a certain degree made wretched thereby.

These are the patients that support our so-called dermatological institutes, that gives fortunes to our non-medical dermatologists, that promote the enormous sale of our sham blood-purifiers. And they are almost all of them curable, and most often by very simple means. Out of the mass of methods that are recommended I propose to detail to you the one that has given me the best results; in other words, the treatment that I can recommend as the routine treatment of acne.

And first the prophylactic treatment, the means to prevent recurrence after the face has been cleared off, and which will suffice for the therapeutic treatment of the mildest cases. This consists more especially in the free use of soap and hot water. There is a prejudice against the use of soap upon the face among the female sex—a prejudice illogical and nonsensical—for there is no complexion beautifier like it. You will make no mistake in ordering your patients to use plenty of good soap and hot water twice daily on the face. The massage opens the sebaceous ducts and expresses the inspissated contents of the glands; it stimulates the muscular structures of the skin; and the hot alkaline water softens and removes the dead epidermis and the dried sebum that clog the mouths of the follicular structures.

Of internal remedies for the acne itself I have but little to say. Most of the drugs recommended for their direct effect on the disease have been found to be absolutely useless. Ergot in good doses may have some effect in stimulating the unstriped muscle of the skin. The arrectores pilorum, attached at one end to the root of the hair, and at the other to the connective tissue of the corium, usually run over the sebaceous glands, and by their contraction assist in the expulsion of their contents. Ichthyol seems to do good in some cases, and, when no other treatment is indicated, it may be administered in 2 and 3 grain doses, thrice daily.

Cod-liver oil is beneficial in cases that show marked tendency to pus formation, with many pustules and dermic abscesses.

Other internal treatment may, however, be important. Whilst

I do not believe that acne is directly caused by derangements of gastro-intestinal or uterine functions, they are undoubtedly of some importance, more especially in prolonging its course. Dyspepsia and constipation in their various forms must be treated on general principles. The diet must be regulated, and usually restricted. Meat should be allowed only once a day; in bad cases a diet of fish fruit, cereals, and light vegetables should be insisted on; and the worst ones require a strictly milk diet for a time. Pastry, sweets, and pickles must be absolutely interdicted. The uterine functions should be attended to, and any irregularity corrected. General hygienic measures are of great importance in every case. An abundant supply of fresh air, proper exercise and bathing must be provided for. An excellent general measure is to order the sponging of the whole body every morning with water as cold as can conveniently be borne, in which salt to the extent of a quarter of a pound to the gallon has been dissolved; to be followed by vigorous friction with a rough Turkish towel.

All these measures, however, are of the nature of adjuvants to the treatment proper of the disease, which is the local one. In a general way this consists in clearing the gland ducts of their plugs of hardened sebum, expressing the contents of the sebaceous glands, stimulating the nutrition of the skin, removing dead and extraneous matter, and generally improving the condition of the organ. And the means that I have found most efficacious for these ends are: First, curetting; second, facial massage; and third, the use of the peeling pastes.

1. The first step in the curettement is to remove all the comedones, and the hard plugs that obstruct the orifices of the sebaceous glands, and cause the formation of the retention cysts with their subsequent follicular and perifollicular inflammation. This is best done with a blunt, spoon-shaped instrument or the metallic handle of one of the ordinary instruments of the pocket case. The comedone extractor sold in the shops, and constructed on the watch-key principle is as inappropriate as that common appliance itself, which unnecessarily wounds the skin, and frequently fails to dislodge the plug. This is effected by gentle lateral pressure. A daily routine of comedo extraction is necessary in the worst cases, and, as it cannot be done by the physician, the patient must be instructed in the method.

Meantime all nodular and pus collections must be thoroughly opened from time to time, either with the little spear-shaped spud that is made for that purpose, or with a tenotomy knife. The exudation and pus is frequently deep seated, and care must be taken to open up the tissues sufficiently to afford exit to the effused material. More extensive infiltrated masses require multiple punctures or cuts, or they may be deeply scarified with a sickle-shaped knife. These measures are beneficial even when no pus is reached; some exudation is always present, a free vent is given to it, and the slightest hemorrhage relieves the perifollicular congestion.

Then follows the curetting, which must be thoroughly and systematically done if results are to be obtained. The curettes themselves are oval metallic sharp-edged instruments set on handles. The operation is commenced with the largest size, the broad edge of the instrument being swept over the skin with an even pressure. The dried and dead epidermis is removed, and the tops of all papules, pustules, and infiltrations are thoroughly torn off—reaccumulation of matter cannot occur—and a free hemorrhage relieves the congested vessels.

This entire process of comedo extraction, puncture of pustules and infiltrations, and curetting must be repeated once in from three to ten days, in accordance with the sensitiveness of the patient's skin. The use of very hot water to the face for a few minutes soon stops the oozing, which may be quite considerable. It is also important that the curettes and knives be sterilized in the flame before they are used, and that the skin of the face be clean, for pus infection not infrequently occurs, and a succession of troublesome and disfiguring dermic abscesses is a not infrequent result of careless curetting.

2. Meantime the patient must employ certain other measures, which may be called auxiliary, but which I regard as just as important as those above mentioned. Once or twice daily, or every other day, in accordance with the reaction, steaming, friction and massage of the face must be performed, and in the following manner: Seated with the head bowed over a bowl of steaming hot water, the patient sponges the face with it by means of a wash rag or thick piece of flannel for some fifteen or twenty minutes. The hot water and the steam soften and macerate the epidermis, remove the dirt and the surface secretion, soften the hardened contents of the sebaceous ducts, and stimulate the muscular structures and the nutritive supply of the skin. The face is then to be vigorously rubbed with the tincture of green soap (green soap, 1 part to 4 or 6 of cologne water), which can be poured on the cloth. This removes the remaining oily matters, clears out the sebaceous glands; and is in turn removed from the face with lukewarm water. The process is completed by anointing the face with a mild sulphur ointment (1 to 10), to replace the oily material that has been lost and to prevent the skin from getting hard and dry. Upon the careful execution of these measures with as much frequency as is possible without causing the skin to become too much inflamed, much of the success of our treatment will depend. And it must be persisted in indefinitely until the tendency to inspissation of the secretion and clogging of the ducts, with the attendant perifolliculitis, is entirely overcome.

3. These measures will suffice for the majority of cases, the milder ones: but many severer examples are met with, in which papules, pustules, and dermic abscesses continue to appear, and more radical means are required. The peeling pastes, so-called, must be employed to cause a desquamation of the epidermis, and remove the accumulated material from the surface. Of the many that may be used I have found that of Lassar most applicable. (B. naphthol 1 part, sulph. præcip. 5 parts, petrolati, sapon. virid., āā 2 parts.) This is best used in the evening, being spread over the face as thick as the back of a table knife and allowed to remain there half an hour, an hour, two hours, or over night, for different skins vary greatly in their sensibility and in the intensity of their reaction to it. As soon as vigorous burning sets in the paste must be wiped off with a soft cloth, and a little cold cream or a boric acid ointment, applied, followed by a dusting powder (zinc, talcum, amylum, *partes equales*). This process must be repeated daily for from four to six days, and until tension and redness of the skin, followed by desquamation, sets in. Then a mild zinc powder or ointment should be used for a number of days, and the peeling process repeated a second or third time, as may be necessary.

This, gentlemen, is the routine treatment that I can recommend for acne, and I am sure that it will give you, as it has done me, satisfaction in the large majority of cases. It will not cure them all; no treatment will do so; and some cases, I am sorry to say, are apparently incurable, at least under the limitations that are imposed upon us in ambulant practice.

37 *West 50th Street.*

